# Periodontal health status, dental caries experience, oral hygiene practices and treatment needs of geriatric tribal population

**DOI:** 10.6026/9732063002001658

**Published:** 2024-11-05

**Authors:** Jyoti Thakur, Sumit Mohan

**Affiliations:** 1 Pedodontics & Preventive Dentistry, Smile Sure Dental and Orthodontic Center, Ranchi, Jharkhand, India; 2 Department of Conservative, Endodontics & Aesthetic Dentistry, Dental Institute, Rajendra Institute of Medical Sciences, Ranchi, Jharkhand, India

**Keywords:** Elderly, health, indigenous, oral hygiene, tribal, periodontitis, dental caries

## Abstract

This pilot project aims to evaluate the oral health condition of Jharkhand's elderly tribal population, which is located in eastern India. There were 200 participants in this descriptive cross-sectional pilot research, 89 of whom were men and 111 of whom were women. The majority of the people in the study group were jobless. Periodontal health, dental cavities and the presence or absence of prostheses were evaluated. The study's findings indicated that older adults (those over 85) had a higher prevalence of periodontal diseases and a greater demand for prostheses. The DMF index revealed that those over 85 years old had a greater frequency of missing teeth and cavitation in the 75-85 age range. The prevalence of oral diseases is quite high among Jharkhand's tribal people. It is concerning that tobacco use is so common. Therefore, to enhance this population's oral health, sufficient education, motivation and training in oral hygiene must be provided.

## Background:

It is believed that human aging is a biological process. Human life expectancy has increased over the last several decades, mostly due to the government's strict enforcement of healthcare regulations and quick medical advances. The World Health Organization (WHO) reports that the elderly population is growing by 2.5 percent per year, compared to the 1.7% yearly growth of the world population. Adults over 80 will account for about 20% of the global population by the end of 2050, making them the demographic group with the highest rate of growth [1]. In India, this has a population of over one billion, the old population over 60 makes up 76 million individuals or 7.6% of the total [2]. Elderly segment is divided into three sub-groups: [3]. A new or young elderly - People aged 65-74 years who tend to be relatively healthy and active; b. Old or mid-old- People aged 75-84 years whose health status varies from healthy and active to those with chronic diseases; c. Elderly population - People above 85 years who might have numerous health issues. To support the health and wellness of the elderly, adequate nourishment is essential. A faster pace of physical and mental deterioration might be caused by inadequate diet. Elderly people's bad eating habits might be exacerbated by poor dental health. A decreased desire or capacity to eat may be caused by painfully loose teeth or poorly fitted dentures. A poor nutritional state threatens the integrity of the mouth cavity [4]. Since missing teeth and/or poorly fitting dentures make it harder to chew and perceive food flavour, dental health is thought to be a significant contributing factor to proper nutrition in the elderly. Mastication is less effective with partial or full dentures than with intact natural teeth, despite chewing efficiency increasing with their usage [4-5]. As a result, dentists are in a unique position to improve the health of the elderly. Dentists should be aware of dietary risk factors in the senior population. Through diligent screening, they may address nutritional issues early on, when they can be most beneficial and successful. The tribal community in India makes up a significant indigenous minority, accounting for 9.01% of the country's overall population, according to 2011 census figures [6-7]. Only 11% of the nation's tribal population is spread out in isolated areas in the southern states, while 82% of the total is concentrated in the central and western regions. The majority of doctors are unaware of the burden of oral diseases, which often results in underestimating and improperly treating the condition. In this context, it would be helpful to know the distribution and prevalence of oral mucosal diseases, periodontal health, *etc.* [7, 8]. There is no trustworthy information on the dental health of elderly people in Jharkhand's tribal populations. Therefore, the current pilot research aimed to evaluate the oral health condition and treatment requirements of Jharkhand's elderly tribal community.

## Materials and Methods:

## Ethical approval:

This study complied with the protocol; ethical approval was obtained from the Institutional Ethical Committee (IEC/Reg No- ECR/769/INST/JH/2015/RR-21) vide Letter No 207 dated 20/9/2023.

## Study setting:

This pilot study is a descriptive cross-sectional household survey conducted in Similiya and Mesra villages of Kanke Block in Ranchi district of Jharkhand, India.

## Study population:

This pilot study was conducted amongst 200 tribal subjects (89 males and 111 females) aged 65 years and above after explaining the study's rationale and seeking written consent from the participants.

## Inclusion criteria:

The following subjects were involved in the study.

[1] Subjects willing to participate in the study

[2] Subjects aged over 65 years

[3] Permanent teeth

[4] Tribal Population

## Exclusion criteria:

The following subjects will be excluded from the study.

[1] Subjects not willing to participate in the study

[2] Subjects below 65 years

[3] Medically compromised patients.

## Methodology:

The respondents were told of the study's goal and asked to provide written informed permission to participate in the interview and dental examination. The WHO Oral Health Assessment form for Adults (2013) was used to conduct the oral evaluation. It includes the participants' demographic, educational and familial information and information on their economic standing, personal habits, general health and medical conditions and dental and oral health conditions. Information on dental care awareness was also tracked. The WHO type III examination, which uses mouth mirrors and CPI probes with added artificial illumination, would be used to assess the treatment requirements independently. The community periodontal index (CPI) was used to evaluate oral health status for periodontal diseases. The DMF Index was used to assess dental caries. Additionally, the prosthesis status was tracked.

## Criteria for measurement:

Assessment of parameters dealing with oral health were assessed by

[1] Obtaining necessary information about the occupation of the study subjects.

[2] Oral hygiene habits.

[3] Adverse habits such as tobacco usage, khaini, bidi *etc.*

[4] Periodontal status of the subjects by using CPITN probe.

[5] DMFT index of the participants

[6] Need for prosthesis.

## Statistical analysis:

SPSS version 24.0 was used to conduct the statistical analysis. The independent t-test and the chi-square test were used to examine all clinical data. To determine if oral health and periodontal state, DMFT index and prosthesis requirement were correlated, Pearson's correlation analysis was used. The P value, or significance, was determined using the two-tailed t-test, where values less than 0.5 were considered statistically significant.

## Results:

The present house-to-house descriptive cross-sectional pilot study was conducted in 2 villages in the Ranchi district on a geriatric tribal population of 200 subjects, out of which 89 were males (44.25%) and 111 were females (55.75%) (Table 1).

## Age groups:

Among 200 study subjects, 93 persons (46.5%) belonged to the age group of 65-74 years, 72 (36 %) were in the age group of 75-84 years and 35 (17.5%) persons were above 85 years (Table 2).

## Occupation:

In our study majority of the study subjects were un-employed which constitutes 121 (60.5%) participants, while 25 participants (12.50%) were farmers, 37 (18.50%) had shops or small businesses and 17 (8.5%) were unskilled labourers (Table 3).

## Oral hygiene habits:

The majority (92.45%) of the study group (n=185) used datun for teeth cleaning and 1.34% of respondents used a toothbrush to maintain oral hygiene. In comparison, only 12 (6.21%) used tooth powder and other cleaning agents (Table 4).

## Tobacco-related habits:

145 subjects had the habit of chewing tobacco and related products such as Gutka, Khaini and Beedi, while 55 subjects had no adverse habits (Table 5).

## Periodontal status:

Maximum bleeding on probing was observed in age groups 65-75 years, while the age group of 85 years and above had a maximum prevalence of periodontal pockets. The mean of presence of calculus was also observed in age 85 years and above. Statistically significant results were observed in all groups while measuring pockets and calculus in all age groups (p<0.05) (Table 6).

## DMFT index:

The mean decayed score was maximum in 75-85 years at 0.89+1.05, while 85-above had the maximum mean missing score at 1.29 + 3.14. However, the results were not statistically significant (p>0.05) (Table 7).

## Prosthetic status:

154 persons had requirement for prostheses (Table 8).

## Discussion:

The most common dental disease in the elderly is caries, caused by aging-related changes in salivary glands; xerostomia brought on by medication use, poor dietary habits and decreased periodontal health and health [10- 9]. It has been shown that older adults have a higher incidence of missing teeth after extractions than younger adults do [10]. The aforementioned research findings were consistent with our own, which showed that the prevalence of missing teeth was greater in the senior population above the age of 85. This might be brought on by reduced mental and physical abilities, resulting in poor oral hygiene care. Globally, dental health is consistently influenced by one's socioeconomic standing. As a person's socioeconomic position declines, oral illnesses in the elderly rise. Rich people are more likely than those in the lower income bracket to be able to pay and get dental treatment [11, 12-13]. Among the most common chronic problems affecting elderly dentate populations are periodontal diseases. Several epidemiological studies have reported age-related increases in periodontal disease frequency and severity. According to Shaju *et al.* [14], periodontitis was found to be more prevalent in India's rural regions than in its metropolitan areas and it was also shown to be more frequent in those who had tobacco-related behaviors. A loss of strength may cause poor handgrip and, as a consequence, poor dental hygiene and periodontitis. As people age, their risk of developing periodontitis and dental caries increases. This might ultimately result in partial or total edentulousness, which can further contribute to nutritional deficiencies because of difficulty masticating food, which causes them to consume a less hard diet [15]. Dental caries that go untreated might eventually result in tooth loss, which is a failure of the dental care system. Edentulism is closely linked to issues with feeding and chewing. Some experts believe it to be a reliable indicator of death, while others link it to a decline in quality of life. Wide variations in the degree of edentulism among nations are evident in epidemiological data on senior populations. These variations may be attributed to variances in the use of dental care, the availability of public financial assistance and/or the adoption of oral health programs [16]. 145 people (72%) of various age groups in our research reported chewing tobacco-related items regularly. These findings are consistent with research in Kalpetta that focused just on senior tribe members 60 years of age and older, finding that 69.3% of them chewed tobacco [17]. The Paniya populace reported extensive usage of paan masala (89.3%) in research by Valsan *et al.* [6]. Of the Santhal tribal people in West Bengal, India, 41.7% of them used different products that included smokeless tobacco and 9.7% of them were habitual smokers as well. One hundred fifty-four participants in our research showed signs of having prosthesis. According to many studies, a minimum of 20 functioning teeth-but only if they are opposing pairs-are required for successful mastication [18-19]. Stomatitis and traumatic ulcers are the most common conditions among people wearing prostheses. These disorders may be made worse by eating poorly and leading unhealthy lifestyles that include smoking, drinking too much alcohol and maintaining bad oral hygiene [20]. Proper maintenance, cleanliness and dental check-ups are crucial for prosthetic wearers to prevent oral health-related health problems.

## Conclusion:

Overall, the elderly population's oral health was typically poor, with high rates of dental caries, periodontal disease and edentulism. These cause problems with mastication, instability of chronic diseases and degradation of oral quality of life, all of which have an immediate impact on an individual's overall well-being and quality of life. Considering that the elderly are ignorant of their dental pathology. Healthcare officials need to put in place programs that teach senior citizens and their caregivers how to practice better oral hygiene and send them to specialists. Dentists also need to be better educated on the significance of dental health for this demographic.

## Figures and Tables

**Table 1:** Age-wise distribution of subjects

**Total Subjects = 200**	**Males**	**89 (44.25%)**
	Females	111 (55.75%)

**Table 2:** Gender wise distribution of subjects

**Age**	**Sample**	**Gender**	**Sample**
65-75 Years	93 (46.5%)	Male	36 (40.44%)
		Female	57 (51.35%)
75-85 Years	72 (36 %)	Male	42 (47.19%)
		Female	30 (27.02%)
85-above	35 (17.5%)	Male	11 (12.35%)
		Female	24 (21.62%)

**Table 3:** Occupation of the subjects

**S. no.**	**Occupation**	**N**	**%**
1	Agriculture	25	12.50%
2	Shop/business	37	18.50%
3	Unskilled/Labourer	17	8.50%
4	Un Employed/Do not Do Anything	121	60.50%

**Table 4:** Oral hygiene practices of the respondents

**Aid**	**N**	**%**
Datun	185	92.45%
Tooth Brush	3	1.34%
Others	12	6.21%s

**Table 5:** Tobacco-related habits of the subjects

**Age**	**Gutka**	**Khaini**	**Bidi**	**Nil**
65-75 Years	17 (18.18%)	22 (23.76%)	34 (36.96%)	20 (21.10%)
75-85 Years	24 (33.33%)	20 (27.72%)	22 (30.43%)	6 (8.52%)
85-above	0 (0.00%)	4 (9.90%)	2 (6.52%)	29 (83.58%)

**Table 6:** Periodontal Status of the Subjects

**Age**	**Bleeding on probing (mean±sd)**	**Calculus(mean±sd)**	**Pockets(mean±sd)**
65-75 Years	2.62±1.63	1.45±1.71	1.07±1.01
75-85 Years	2.37±1.72	1.24±1.47	0.92±0.90
85-above	1.70±2.36	2.29±2.14	1.14±0.90
P value	0.41(Not Significant)	0.032(Significant)	0.002(Significant)

**Table 7:** DMF index of the participants

**Age**	**Decayed (mean±sd)**	**Missing (mean±sd)**	**Filled (mean±sd)**
65-75 Years	0.51±0.86	0.30±2.15	0
75-85 Years	0.89±1.05	0.12±1.11	0
85-above	0.30±0.78	1.29±3.14	0
P value	0.4(Not Significant)	0.5(Not Significant)	

**Table 8:** Need for prosthesis in subjects

**Age**	**N**	**Prosthesis present**	**Prosthesis required**	**Tooth**
65-75 Years	93	18 (19.35%)	65 (69.89%)	Maxillary=12, Mandibular=39, Both=14
75-85 Years	72	05 (6.94%)	54 (75.00%)	Maxillary=11, Mandibular=17, Both=26
85 Above	35	0 (0.00%)	35 (100%)	Maxillary=8, Mandibular=15, Both=12
